# Understanding the Multidimensional Nature of Student Engagement During the First Year of Higher Education

**DOI:** 10.3389/fpsyg.2019.01056

**Published:** 2019-05-10

**Authors:** Vesa Korhonen, Markus Mattsson, Mikko Inkinen, Auli Toom

**Affiliations:** ^1^Faculty of Education and Culture, Tampere University, Tampere, Finland; ^2^Centre for University Teaching and Learning, Faculty of Educational Sciences, University of Helsinki, Helsinki, Finland

**Keywords:** engagement, higher education, retention, first year experience, network analysis (NA)

## Abstract

In the description of the complex relationship between individual students and their education context, as well as understanding of questions related to progression, retention or dropouts in higher education, student engagement is considered the primary construct. In particular, the significance of the first year of higher education in terms of engagement is decisive. We aim at developing a multidimensional conceptualization of engagement and utilized network analysis. Data were collected as part of the annual Student Barometer survey in Finland during the 2012–2013 academic year, and we gathered a nationally representative sample (*n* = 2422) of first-year students in different disciplines at 13 Finnish universities. Network analysis confirmed the multidimensional process model of engagement and its six dimensions. The central dimensions of engagement are identity and sense of belonging, which develop in the interplay between individual and collective dimensions as a long-term process. Additional network analyses with covariates identified positive and negative factors that affect engagement. The study adds new perspectives to existing knowledge of engagement. It is important to understand the process-like nature of engagement and make visible factors affecting the process. Based on these findings, we provide novel practical recommendations for interventions for university students who struggle with engagement during their first year.

## Introduction

In the focus on the interaction between university students and their education environment, as well as development of ways of conceptualizing and measuring this process in different disciplinary contexts, student engagement has become an important perspective (Koljatic and Kuh, 2001; Handelsman et al., 2005; Langley, 2006; Leach and Zepke, 2011; Kahu, 2013). In descriptions of the complex relationship between individual students and their educational context, as well as understanding questions related to progression, retention or dropping out of school, engagement is considered the primary construct (Trowler, 2010; Christenson et al., 2012). Strong student engagement has been shown to be linked to smooth progression of studies, positive learning experiences, a deep approach to learning, general satisfaction, well-being and persistence, as well as better learning outcomes, such as quality of knowledge, higher-order thinking, ethical qualities, career readiness and intentions, professional identity and grades (Zhao and Kuh, 2004; Scott, 2008; Harper and Quaye, 2009; Trowler and Trowler, 2010; Kahu, 2013; Millard et al., 2013).

The first year of higher education has been identified as a crucial phase from the viewpoint of successful engagement (Krause and Coates, 2008) and influences a student’s educational career. First-year students familiarize themselves with their domain and the practices of their scholarly learning community (Lave and Wenger, 1991; Macaskill and Taylor, 2010). Students’ previous experiences, motives for studying and abilities to adapt themselves to new practices, as well as the atmosphere and participatory qualities of the community, affect the successful ongoing transition to and engagement in the community (Tinto, 2003; Zhao and Kuh, 2004). The consequences of disengagement are serious, because they might lead to student attrition, unplanned changes in the study program, withdrawal and even failure to complete one’s education (Heirdsfield et al., 2008). Attrition rates are significantly higher during the first year of higher education, and thus, student engagement with the scholarly learning community is necessary (Krause et al., 2005). Such understandings respond to the multifaceted and complex needs of diversifying student populations in present-day universities (Harper and Quaye, 2009).

Engagement emerges in the interaction between the student and the institution (Astin, 1984, 1993; Pascarella and Terenzini, 2005; Kuh, 2008). In this dynamic, individual objectives and starting points for engagement, as well as empowering contextual elements, are essential. Previous studies have shown that internal factors (like motivation, expectations for higher education and emotions) and formal and informal external contextual factors (like systemic structures, curricular issues, and pedagogical practices) contribute either positively or negatively to student engagement (Tinto, 2000; Pascarella and Terenzini, 2005; Lardner and Malnarich, 2008; Harper and Quaye, 2009; Matthews et al., 2011; Poutanen et al., 2012; Soria and Stebleton, 2012; Kahu, 2013).

Previous research on student engagement covered multiple different perspectives, from formal and informal aspects of student experience (Coates, 2007) to the intrinsic or extrinsic dimensions of experiences of engagement (Fredricks et al., 2004; Trowler, 2010; Kahu, 2013). However, studies focused simultaneously on the internal and external factors that contribute to student engagement that take into account the dynamics between individuals and the context are scarce. The primary key factors in first-year student engagement have not been systematically identified. Moreover, individual differences and variation in students’ engagement since the beginning of their education have not been widely explored. Therefore, this study explores the structure of student engagement and the dynamics of intrinsic and extrinsic factors related to student engagement and utilizes network analysis.

### Theoretical Conceptualization of the Engagement Process

Most of the established conceptualizations of student engagement are based on the conception that engagement emerges in the interaction between the student and the educational context (Astin, 1993; Pascarella and Terenzini, 2005; Kuh, 2008; Kahu, 2013). A growing body of evidence suggests that participating in learning communities contributes positively to student engagement, which, in turn, may affect educational attainment and smoother progression of education (Pike et al., 2011, 2012). The effect can be seen in the purposeful activities that strengthen the engagement (Kuh et al., 2006; Kuh, 2008), especially community-based practices, such as learning communities through curricula and courses, common assignments and projects, and students working together with experienced scholars (Kuh et al., 2006; Kuh, 2008). Zhao and Kuh (2004) found that the relationship between membership in learning communities and student engagement is significant especially for first-year students. Strongly engaged students tend to emphasize the meaning of social relations and cooperation for their education (peer communities and the academic teaching–learning community; Coates, 2007).

**FIGURE 1:**
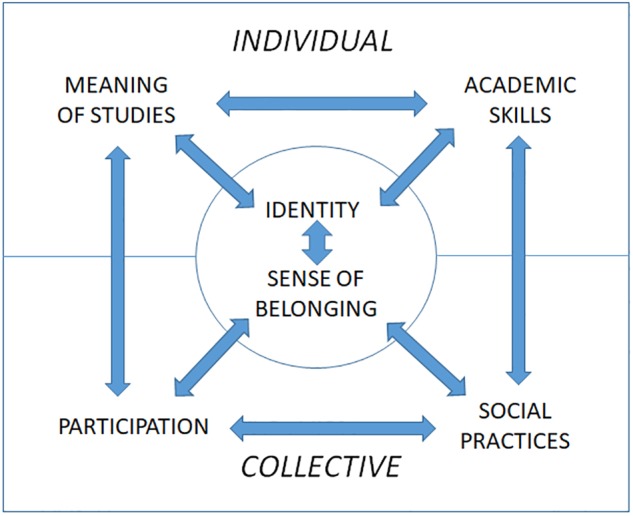
Model for student engagement.

From the viewpoint of Wenger’s (1998, 2010) situational and sociocultural theorization of community of practice, strong student engagement means emerging bonds between the student and the closest discipline-related communities. For successful engagement, two intertwined processes must be realized: student’s self-motivated active agency (subjectivity) and developed and deepened participation in discipline-related communities (collectivity; Wenger, 1998). Engaging experiences may occur during the participation processes for educationally effective and inclusive practices (Coates, 2007; Harper and Quaye, 2009; Leach and Zepke, 2011). Following Wenger’s thinking, we defined four core areas for the engagement process (meaning, participation, sense of belonging and sense of identity) and complemented the overall picture with two more dimensions: academic skills and social practices (see Figure 1). Academic and social integration in studies play a fundamental role, for example, in widely cited Tinto’s (1993, 2003) model. The *individual process* consists of experiencing one’s education meaningfully together with mastering certain central academic skills, whereas the *collaborative process* consists of participating in academic teaching–learning communities and adopting certain social practices. In our view, student engagement is constructed based on these processes. The fundamental features of the engagement process, “sense of belonging” and an evolving “identity,” emerge from the interaction of individual and collective processes.

The dimensions of engagement were first outlined in a conceptual article (Korhonen, 2012) and next a theory-driven qualitative study with a sample of university students (Poutanen et al., 2012) was conducted, where the dimensions were found appropriate for describing students’ experiences of engagement. The term “meaning of studies” is used here to refer to the personal significance of the recently started education program and the perceived opportunities higher education offers to the student. This dimension covers students’ self-concepts, values, attitudes or beliefs regarding their education (Kim and Sax, 2014). Students bring their own meanings and preferences to the educational experience, and this has been shown to affect academic motivation and values (Henderson-King and Smith, 2006). “Academic skills” refer to the skills that are necessary for participating in academic teaching–learning practices. Chickering and Reisser (1993) used the similar concept “academic competence” which involves knowledge acquisition, increased intellectual sophistication and development of higher-order cognitive skills.

Turning to collective aspects of engagement, by “participation” we refer to taking part in different study-related communities, such as student peer communities or academic teaching–learning communities. Wenger (1998) defines participation as a process of being in relationships with others. It suggests action and connections in local communities of practice. The term “social practices” is used to refer to the various disciplinary practices through which students become socialized in a disciplinary culture (Becher and Trowler, 2001; Trowler, 2008). Students do not simply learn “about” something; instead, they also learn “to be” and “to do” something. The social practices perspective takes learning as an aspect of participation in socially situated and locally constructed practices (Korhonen, 2012). Scholars have shown that engagement varies considerably among students in different disciplines (Brint et al., 2008; Kahu, 2013). Therefore, locally constructed practices play an important role in triggering engagement.

From the viewpoint of engagement, “identity” refers to how learners interpret their experiences, perceive their actions and function as active agents in an academic environment (Chapman and Pyvis, 2005; Briggs et al., 2012). The developing identity refers to the students’ personal insight into themselves and their abilities as learners, as well as the ways in which they position themselves in various communities related to their education (Chapman and Pyvis, 2005). When students experience their education as meaningful for the goals they have set for themselves, the feeling of belonging is strengthened (Tinto, 2000). An appropriate definition for the sense of belonging consists of a student being accepted, valued, included and encouraged by others, such as teachers and peer learners (Thomas, 2012; Toom et al., 2017). Students’ positive experiences and actual patterns of participation naturally affect the students’ developing sense of belonging (Lester et al., 2013; Masika and Jones, 2016). Together with a student’s developing professional identity as a member of the academic community, the sense of belonging makes up the overall process of engagement. Engaging experiences create a stronger sense of belonging that further expands identity in multiple different ways (Wenger, 1998).

### Factors Intertwined With Student Engagement

From the viewpoint of the integrative theorization of student engagement in this study, it is interesting to investigate how students’ motivations to study, approaches to learning, chosen field of study and possible intention to drop out are intertwined. These are relatively established theoretical constructs and fields of research, and they at least partly overlap with academic and social, as well as individual and collective, aspects of student engagement—or disengagement. The motives for attending higher education are related to the skills necessary for education (Coté and Levine, 1997, 2000). According to Coté and Levine (1997, 2000), these motives are categorized into five basic dimensions. *Personal-intellectual development* means interest in intellectual and cultural self-development and a striving for understanding the complexities of life. *Humanitarian* motivation means an internal interest in improving the world, changing the system and helping others. *Expectation-driven* motivation refers to a student’s efforts to meet the expectations of family and friends to attend university and obtain a degree. *Careerism–materialism* means seeing the degree as a tool for achieving a certain social and economic status in life. *Default motivation* refers to a situation in which students do not really know why they are attending higher education, just that they consider it a better option than the alternatives. Previous studies with Canadian and Finnish university students showed that these study motives are connected to the nature of students’ engagement and progression in education (Coté and Levine, 1997, 2000; Korhonen and Rautopuro, 2012; Saari, 2013). Specifically, Coté and Levine (1997) showed that personal-intellectual motivation predicted the development of good self-management and self-motivation skills, while default motivation was related to a poorer prognosis for skill development and academic achievement.

University students adopt different approaches to learning depending on the task at hand, their skills and strategies, as well as the characteristics of the learning environment (Marton and Säljö, 1976; Entwistle and Ramsden, 1983). Students with a *surface approach to learning* tend to memorize facts and reproduce information, and as a result, have fragmented knowledge (Entwistle and Ramsden, 1983; Lindblom-Ylänne et al., 2018). Students who apply the *deep approach* have intention to analyze and understand and thus, utilize multiple strategies in their learning to evaluate and relate the contents to be studied (Entwistle and Ramsden, 1983). The *strategic approach* focuses on students’ intention to achieve the highest grades and especially, on their method for regulating their studying effectively (Entwistle, 2009). A previous study viewed the deep approach to learning as a component of cognitive engagement, and it is at least partly a matter of definition whether approaches to learning and engagement are different or overlapping phenomena (Korhonen et al., 2017).

Intention to drop out has been found to be related to challenges experienced in education and to weak engagement, which can further be seen to be connected to slow progress in education (Korhonen and Rautopuro, 2018). Specifically, Korhonen and Rautopuro (2012) found expectation-driven motivation and default motivation predict problems in adapting to one’s program and motivating oneself to study or the lack thereof, in the case of personal-intellectual motivation. Intention to drop out can be related either to social or academic aspects of studies, or reasons can be found outside the department, for example, due to changes in life and work situations. Intention to drop out can develop for many different reasons, and the intention tends to evolve slowly. They are serious indications of problems in the students’ well-being and engagement, and they are associated with students’ self-regulation skills, interactions in the study-related communities and academic experiences in their program (Kahn, 2014; Natoli et al., 2015). In other words, intention to drop out shows students’ overall disengagement with their education and the communities in which the educational programs take place.

### Network Analysis as a Methodological Basis for Studying Engagement

We perceive engagement as a dynamic phenomenon that emerges from the complex interactions of the components. For instance, interactions among people are classic examples of systems that can be modeled as networks where nodes correspond to people, and edges connecting nodes correspond to the nature of the relationship. Recently, the idea of networks was applied to descriptions of psychological phenomena, such as depression (Fried, 2015; Fried et al., 2017), posttraumatic stress disorder (PTSD; McNally et al., 2015), intelligence (Van Der Maas et al., 2006) and health-related quality of life (Kossakowski et al., 2016). In psychopathological network models, individual symptoms figure as the nodes. This is in marked contrast to the traditionally employed latent variable models in which the components of psychological characteristics are seen as passive reflections of the underlying constructs. Further, although latent variable models are premised on the idea that the components are interchangeable, in network models the centrality or importance of the components can be assessed (McNally et al., 2015). Therefore, we think that network analysis is well suited for describing how the phenomenon of engagement is formed when its components influence each other in complex ways.

In psychometric network models, the term “component” refers to a part of the network that bears unique causal relations to the rest of the network (Cramer et al., 2012; Borsboom, 2017). For instance, perceiving one’s education as meaningful has a cognitive component (M1, perceiving one’s education as supporting self-development) and an emotional component (M2, being enthusiastic about education) that are differentially related to the remaining components of engagement. Accordingly, we interpret the study findings as causal hypotheses, while bearing in mind that (1) conditioning on a common effect of two variables may introduce a spurious edge into the graph (Epskamp et al., 2018; Rohrer, 2018), (2) a clique in the network graph may indicate the presence of an unmodeled latent variable (Epskamp et al., 2018) and (3) the direction of the potential causal effect cannot be inferred from the network graph (Epskamp et al., 2018).

Background variables or outcome variables can be included in a network model (McNally et al., 2015). We adopt the idea and assess the relations between the components of engagement and motives for attending university, students’ approaches to learning, their intention to drop out of their program and students’ certainty about their chosen field of study. These factors have been proven to be central for successful engagement, or the opposite, for disruptive engagement in the first year of higher education. For instance, problems in self-regulation and management of one’s own learning are obvious among disengaged and slowly progressing higher education students in Finland (Korhonen and Rautopuro, 2012). Furthermore, uncertainty about chosen field of study and intention to drop out are typical of this disengaged group. When examining connections to motives for attending university, problems managing learning correlate in particular with the default motivation and somewhat with the expectation-driven motivation (Korhonen and Rautopuro, 2012). These factors related to study motives, students’ approaches to learning, and their intention to drop out form a complex set of intertwined factors. It has also been observed that dropping out of school is most common during the first year of higher education (Rautopuro and Väisänen, 2001; Korhonen and Rautopuro, 2018). Therefore, this phase is crucial for the education career as a whole. Further, from the perspective of beginning higher education, and continuing, it is crucial that students feel that their chosen field of study is right for them. In contrast, the unstructured default study motivation seems to lead to problems in managing one’s learning and to intention to drop out (Korhonen and Rautopuro, 2012).

### Aim and Research Questions

This study used network analysis to gain a better understanding of the intrinsic (individual/psychological) and extrinsic (collective/contextual) components of student engagement and the complex associations between these aspects. Based on previous research, we developed a student-centered method of measuring engagement in different disciplinary contexts. To identify the key components and structure of engagement, the following research questions are addressed:

(1) How do the core components of engagement interact to give rise to the phenomenon of engagement?
(2) How are the components of engagement related to
  (a) students’ motives (especially personal-intellectual and default motives) for attending the university?
  (b) students’ approaches to learning?
  (c) whether students intend to drop out?

## Materials and Methods

### Participants, Design, and Data Collection

The target population of the study were the all first-year students in different disciplines at 13 Finnish universities. The aim was to collect a nationally representative sample of the Finnish first-year university students. The sampling procedure was designed in co-operation with the Finnish Research Foundation of Studying and Education (OTUS), which also conducted the data collection in connection with the Finnish Student Barometer survey (Saari and Kettunen, 2013). The final target population was 16,972 (where men 42.5% and women 57.5%). This describes the gender distribution of Finnish university students where majority are women, like in studied year in target population (43.0% men and 57.0% women: Official Statistics of Finland [OSF], 2019). From the target population, about one-third were randomly chosen to the sample population for the study. Because of a small sampling error, medical students were excluded from the final sample and the remaining sample population size is 6,040 (where men 42.5% and women 57.5%), as mentioned in Table 1.

**Table 1:** The target population, the sample population and the actual participants by discipline.

Academic field	New students 2012	Sample population	% of new students	Participants	Response %
Agricultural sciences	362	233	64%	96	41.2%
Arts	413	139	34%	56	40.3%
Business and management	2216	495	22%	139	28.1%
Educational sciences	2141	815	38%	327	40.1%
Engineering and technology	2539	589	23%	173	29.4%
Pharmacy	366	161	44%	73	45.3%
Health sciences	436	148	34%	72	48.6%
Humanities	2408	940	39%	454	48.3%
Law	561	295	53%	117	39.7%
Natural sciences	3118	1200	38%	431	35.9%
Psychology	218	94	43%	59	62.8%
Social sciences	1846	697	38%	316	45.3%
Theology	279	179	64%	72	40.2%
Veterinary medicine	69	55	80%	37	67.3%
Total	16972	6040	36%	2422	40.1%


The University of Helsinki was weighted in the sample population because there were aims to use collected data in their own development work, but otherwise, each first-year student from each university and each discipline had the same probability of being included in the sample population. The survey was targeted at Finnish students and was implemented in Finnish; therefore, respondents from the majority population were emphasized. The share of international students in Finnish higher education at the time of the survey was 9.7% of all students (CIMO, 2014). Because the largest university in Finland, the University of Helsinki, was emphasized in the sample, less common academic fields, such as veterinary medicine, theology and agriculture and forestry, were slightly overrepresented in the sample population.

The final sample, those who participated in the survey during the academic year 2012–2013, comprised altogether 2,422 first-year students [men: 574 (23.7%); women: 1,848 (76.3%)]. They ranged in age from 19 to 67 years (mean: 24.1 years, median: 22.0 years; Table 1). Almost all participants (98.1%) were Finnish citizens. The final response rate was satisfactory 40.1%.

Data for this study were collected with an extensive online questionnaire (Saari and Kettunen, 2013), which included background information, previous education, application motives, education progress, first-year experiences, values, attitudes, well-being, subsistence and employment. The authors of this study suggested three additional specific areas for the online questionnaire: student engagement, motives for attending university and learning approaches. These were utilized in this study. The students’ participation in the study was voluntary, and the participants’ informed consents were guaranteed. Informed consent was inferred from the participants’ returning of the questionnaires. The study did not include any threat to physical integrity, children under the age of 15, strong stimuli, mental harm or the risk of safety for participants (cf. ethical principles of research in the humanities and social and behavioral sciences and proposals for ethical review, prepared by the Finnish Advisory Board on Research Integrity, 2009). According to the principles, this study did not require ethical review and approval in Finland.

### Measures

For this study, we utilized the following measures and scales included in the 2013 Finnish Student Barometer survey [Saari and Kettunen, 2013: the Engagement Evaluation Questionnaire (EEQ; Korhonen et al., 2013, 2017), the Student Motivations for Attending University (SMAU) questionnaire (Coté and Levine, 1997, 2000) and Learning Strategy scales from the HowULearn questionnaire (Parpala and Lindblom-Ylänne, 2012)]. In addition, questions about intention to drop out and chosen field of study were included.

#### Engagement Evaluation Questionnaire

The EEQ was used to measure the different dimensions of the student engagement process (Korhonen et al., 2013, 2017) and was utilized as the main measure to understand the multidimensionality of student engagement during the first year of higher education. The EEQ was developed to measure the theory-based dimensions of student engagement and aimed at getting an overall picture of the three overlapping engagement processes: individual, collaborative, and engaging (Korhonen, 2012). These processes are operationalized into six subscales: meaning (M), academic skills (Sk), participation (Pa), social practices (Pr), sense of belonging (B), and identity (I). All items on these subscales were measured with a seven-point Likert scale (1 = strongly disagree, 7 = strongly agree). The EEQ was applied in a 12-item version form in this study (six subscales, two items on each subscale). The psychometric properties of the EEQ measurement from the point of view of classical test theory has been demonstrated in our previous study concerning transitions between first and second year in university education (Korhonen et al., 2017), where Cronbach alpha test values for subscales in both years were for meaning (M) 0.82–0.83, academic skills (Sk) 0.70–0.73, participation (Pa) 0.75–0.75, social practices (Pr) 0.50–0.64, sense of belonging (B) 0.69–0.76 and identity (I) 0.74–0.80.

#### Student Motives for Attending University

Student motives for attending university and their possible connections to the engagement dimensions were an area of interest in this study. SMAU is a questionnaire that has five scales measuring different motives for attending university: personal-intellectual development, humanitarian, expectation-driven, careerism–materialism and default (Coté and Levine, 1997, 2000). The original SMAU questionnaire consists of 23 items (Coté and Levine, 1997), and it resembles other widely used student typologies (i.e., Astin, 1993). Coté and Levine (1997) stated that their typology better reflects attitudes and motivations formed before university participation. We utilized a shortened 15-item version of the SMAU questionnaire, where the three top-selective items were chosen for each of the five subscales. The subscales describing different motives for attending university are personal-intellectual development, humanitarian, careerism–materialism, expectation-driven and default motivation. All items in these scales were measured in this study with a seven-point Likert scale (1 = strongly disagree, 7 = strongly agree). The SMAU questionnaire has been tested to be a reliable instrument in previous Canadian (Coté and Levine, 1997, 2000) and Finnish studies (Korhonen and Rautopuro, 2012; Saari, 2013) concerning university students.

#### Approaches to Learning

We also observed the use of learning strategies and their relationship to the different areas of student engagement in the first year of higher education and adopted three appropriate scales (deep, systematic and surface) from the HowULearn questionnaire used previously with Finnish university students (Parpala and Lindblom-Ylänne, 2012). We measured the approaches to learning on a short two-item form in each of the three learning strategy scales. The items on the scales were measured with a seven-point Likert scale (1 = strongly disagree, 7 = strongly agree). The validity and reliability of these scales were demonstrated in previous studies in different learning contexts (see Parpala et al., 2013).

### Analysis Methods

#### Network Analysis

When network models are used as psychometric models, the key difference from the traditional application areas of network analysis is that the network weights are parameters whose values are estimated from the data (Epskamp et al., 2017). After the estimates have been calculated, traditional methods of characterizing networks, such as different centrality indices, can be calculated the same way as for other network models. Network weights in psychometric models have been estimated by calculating either correlations or partial correlations (Epskamp et al., 2017), resulting in both cases in a signed and weighted network model. However, it has become customary to analyze data in the form of partial correlations (Epskamp et al., 2017), as pairwise associations, when controlling for the effects of the other variables, are the phenomenon of central interest in network models. A non-zero edge may indicate potential causal connections (Epskamp and Fried, 2018), logical relationships among the nodes (Kossakowski et al., 2016), while the possibility remains that a cluster of nodes is formed because of an unmodeled latent variable influencing all the nodes in the cluster (Golino and Epskamp, 2017).

Partial correlations are often estimated using a method known as the graphical lasso, which constrains small correlations to zeroes (Epskamp et al., 2017), thus avoiding capitalizing on sampling variation and results in what are known as sparse models. In this contribution, the level of sparsity was determined by the tuning parameter λ, the value of which was chosen based on the extended Bayesian information criterion (EBIC; Foygel and Drton, 2010). The EBIC model selection is governed by the hyperparameter γ, which was set to the recommended default value of 0.5 (Foygel and Drton, 2010). The locations of the nodes were determined using a modified version of the Fruchterman and Reingold (1991) algorithm for weighted networks (Epskamp et al., 2012), which places strongly connected nodes that have many edges in common close to one another. All network analyses were performed in R using the packages qgraph (Epskamp et al., 2012) and bootnet (Epskamp et al., 2017).

#### Centrality Indices in Network Models

We used three centrality indices to characterize the networks: strength, betweenness and closeness centrality (Opsahl et al., 2010). The first is defined simply as the sum of the absolute values of the weights of the edges connected to the focal node, and thus, this index describes the extent that a node is connected to other nodes. The two other centrality indices are defined using the concept of distance between the nodes in a network. In a weighted network, the distance between two nodes is defined as the inverse of their connection weight. Betweenness centrality is the distance of the focal node to other nodes, and it quantifies the importance of a node in connecting other nodes of the network. Closeness centrality is the inverse of the average distance of the focal to node to other nodes in the network, and it quantifies the degree to which the node is indirectly connected to other nodes in the network. For a succinct introduction to the centrality indices, please see Costantini et al. (2015).

#### Accuracy and Stability of the Results

To assess the potential replicability (the stability and accuracy) of the results, we performed bootstrap analyses in which we calculated the confidence intervals for the edge weights and the centrality indices. The bootstrap analyses help assess the degree to which the results are affected by sampling variability. This procedure is described by Epskamp et al. (2017).

In assessing the *accuracy* of the edge weight estimates, we used non-parametric bootstrapping (resampling the data with replacements) because most of the input variables are ordinals. Further, we assessed the *stability* of the centrality indices using the so-called *case-dropping subset bootstrap*, which involves repeatedly calculating the values of the centrality indices based on different subsets of data. If the results depend on carrying out the analyses specifically on the original sample, then they cannot be considered stable. Stability is assessed using the correlation stability coefficient (CS coefficient), which indicates the maximum proportion of cases that can be dropped such that with 95% probability the correlation between the original centrality indices and those calculated based on the subset exceeds 0.7 (Cohen’s suggested value indicates a very large effect) (cf. Cohen, 1977; Epskamp et al., 2017). The values of the CS coefficient should, at a minimum, exceed 0.25, and preferably be larger than 0.5 (Epskamp et al., 2017).

We assessed the differences between the values of the edge weights and the centrality indices by calculating bootstrapped confidence intervals for the difference scores of all pairs of edge weight or centrality index values. If the bootstrapped confidence interval for the difference score does not cover the value of zero, the edge weights or the values of the centrality indices can be said to differ from one another (Epskamp et al., 2017). No correction for multiple comparisons was made (for a discussion of the problematicity of performing such corrections in this context, see Epskamp et al., 2017). This procedure is called the bootstrapped difference test.

## Results

### Engagement Phenomenon in the Total Sample of First-Year Students

Descriptive statistics related to the engagement items on the EEQ are displayed in Table 2, which shows that network analysis is a feasible option for these data: The item means are not extremely high or extremely low, and all variables exhibit roughly similar amounts of variability. As described, the engagement items and dimensions were operationalized into six subscales on the EEQ, including two items in each subscale: meaning (M), academic skills (Sk), participation (Pa), social practices (Pr), sense of belonging (B), and identity (I).

**Table 2:** Descriptive statistics of the engagement items and the covariates.

Item	Mean	SD	Skewness	Kurtosis
M1 (self-development)	5.75	1.18	–1.27	2.37
M2 (enthusiasm)	5.53	1.36	–1.00	0.86
Pa1 (not knowing others)	2.17	1.63	1.43	1.14
Pa2 (contacts with others)	5.04	1.62	–0.76	–0.13
Pr1 (working in small groups)	3.54	1.78	0.26	–0.97
Pr2 (education as a solitary enterprise)	4.55	1.59	–0.35	–0.67
Sk1 (scheduling)	3.69	1.70	0.19	–0.92
Sk2 (regular studying)	4.75	1.55	–0.57	–0.34
I1 (fit in well as a university student)	5.48	1.29	–0.85	0.53
I2 (have found an appropriate study method)	4.75	1.41	–0.41	–0.31
B1 (belongingness)	5.40	1.41	–0.93	0.64
B2 (alienation)	2.27	1.48	1.19	0.76
Personal-intellectual motivation	5.70	1.06	–1.08	1.52
Default motivation	2.45	1.35	0.83	–0.05
Certainty about field of study	1.74	0.44	–1.11	–0.76
Intention to drop out	1.26	0.44	1.07	–0.87
Deep approach	5.15	1.02	–0.41	0.19
Strategic approach	4.20	1.38	–0.03	–0.62
Surface approach	3.61	1.29	0.17	–0.30


Meaning (M) and academic skills (Sk) represent the individual process of engagement. The two items related to experiencing one’s education as meaningful are M1 (self-development) and M2 (enthusiasm), while the two items related to academic skills (Sk) are Sk1 (scheduling) and Sk2 (regular studying). Participation (Pa) and social practices (Pr) represent the collaborative process of engagement. The items related to participation are Pa1 (not knowing others) and Pa2 (contacts with others), while the two items related to social practices are Pr1 (working in small groups) and Pr2 (education as a solitary enterprise). Finally, sense of belonging (B) and identity (I) represent the overarching properties of the engagement process. The items related to sense of belonging (B) are B1 (belongingness) and B2 (alienation), while the items related to identity are I1 (fit in well as a university student) and I2 (have found an appropriate study method). From Suppelementary Tables S1 and S2 more information on inter-item relations.

**FIGURE 2:**
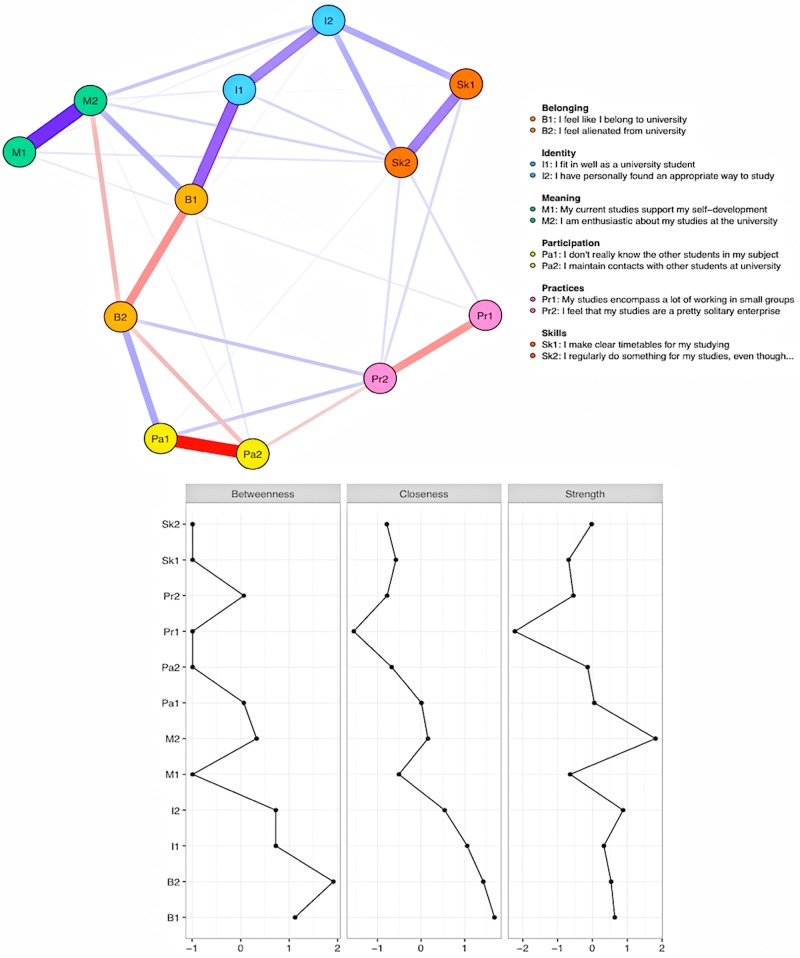
Network of student engagement (top) and the associated centrality indices (bottom).

The lasso-estimated partial correlation network of the 12 engagement items is shown in Figure 2 with the associated centrality indices. The colors of the nodes were chosen to reflect the composition of the phenomenon of engagement. The strength of the partial correlations among the components of engagement is reflected in the width and saturation of the edges connecting the nodes with blue edges corresponding to positive associations and red to negative ones. For instance, an extremely strong connection between the two meaning items (M1 and M2) remains when controlling for the other connections. The edge weights were estimated accurately as evidenced by the narrow confidence intervals in Supplementary Figure S1. This enables us to interpret differences among the edge weights. The values of the centrality indices were similarly stable for changes in the composition of the sample (Supplementary Figure S2). The centrality index values remained extremely stable even when up to 70% of the original cases were dropped. The CS index values were similarly high for all three centrality indices, CS (cor = 0.7) = 0.75 for all three indices. These results show that all three centrality indices are interpretable as they stand.

The centrality indices in the lower part of Figure 2 indicate unequivocally that the most central components of engagement were the experiences of belonging and alienation (nodes B1 and B2) and assuming the role of a student (nodes I1 and I2). The bootstrapped difference tests (Supplementary Figure S3) showed that the centralities of these nodes differed for the most part from the rest of the network but were very similar to each other. The high closeness centralities indicate that all other nodes can be easily reached from these four nodes via direct or indirect paths. Changes in the closeness centrality nodes had a marked effect on other nodes in the network. Nodes B2 (alienation) and B1 (belonging) had the highest betweenness centralities in the network. In this model, the alienation node links the nodes related to participation (Pa1 and Pa2) and social practices (Pr2) to the rest of the network through belongingness (B1) and enthusiasm (M2). Finally, being enthusiastic about one’s education (M2) had the highest strength centrality in the network (Supplementary Figure S3), and thus, was related to many other parts of the engagement phenomenon whereas there are only very weak links from M1 (self-development) to the rest of the network.

### Analyses With Covariates

**FIGURE 3:**
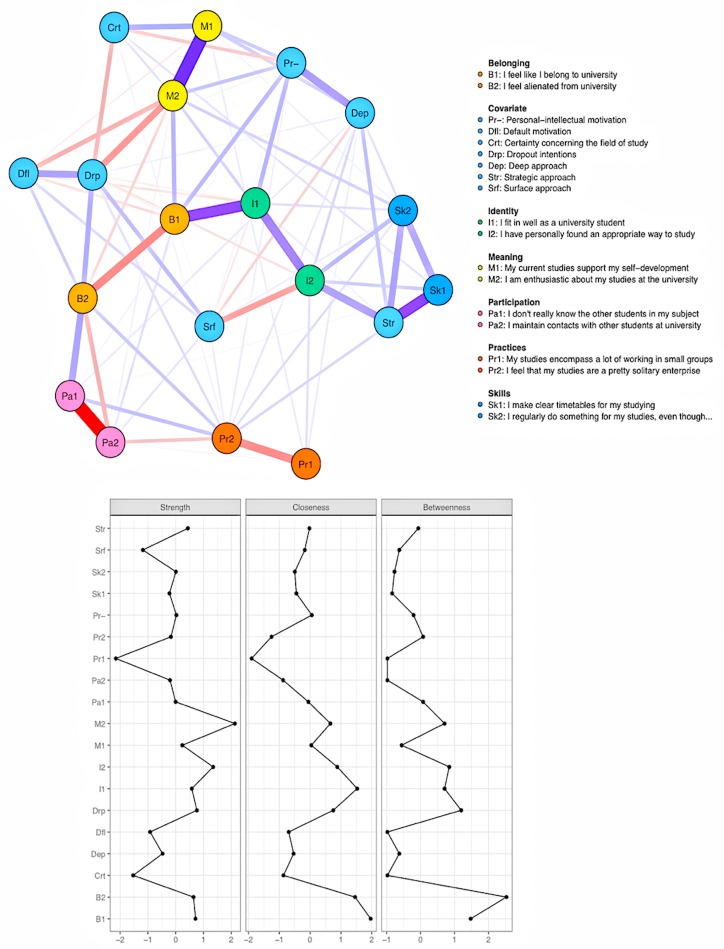
Network of student engagement and covariates (top) and the associated centrality indices (bottom).

In addition to the network analysis of student engagement, we investigated the ways that the students’ motives for education, approaches to learning and intention to drop out were related to students’ engagement in education. The analysis was exploratory: Based on the evidence presented in the Introduction, we assumed that the students’ motives to study would be related to engagement, although we could not derive exact hypotheses from previous research. Therefore, we first performed an analysis that included all five motives and approaches to learning, intention to drop out and students’ certainty about their chosen field of study. These preliminary analyses are reported in the Supplementary Figures S4–S7. The analyses showed that of the five motives, the default motivation and personal-intellectual motivation were linked to various components of engagement. Thus, and for the sake of simplicity, we report the network model incorporating these motives in Figure 3.

The edge weight estimates were very accurate as indicated by the narrow confidence intervals in Supplementary Figure S8. The estimates of the values of the centrality indices were similarly stable (Supplementary Figure S9), with the CS index was 0.75 for all three indices.

Personal-intellectual motivation was positively associated with the components of engagement that were deemed central in the model shown in Figure 2 (B1, feeling like belonging to the university, and I1, fitting in well as a university student). Similarly, having a deep approach to learning and perceiving education as supporting self-development shared positive links with personal-intellectual motivation. Interestingly, the more certain the students were about their field of study, the lower personal-intellectual motivation they had. Default motivation, however, was related to considering dropping out of school and feeling alienated from the university (B2) and shared a negative link with being enthusiastic about one’s education.

When the associations that the approaches to learning had with the rest of the network were examined, the deep approach was positively associated with personal-intellectual motivation as observed above. Personal-intellectual motivation also had quite a strong positive association with the strategic approach, which, for its part, was most strongly connected with the nodes related to study skills (Sk1, Sk2, and I2). The surface approach was related to intention to drop out and not having found an appropriate study method.

Last, intending to drop out from one’s university was related, through a negative edge, to certainty about one’s field of study and being enthusiastic about one’s education. Further, this node shared a positive edge with default motivation, feeling alienated from the university and the surface approach to learning. Interestingly, intention to drop out emerged as the third most strength central node in the network, signifying that this node had strong connections to the rest of the network. Intention to drop out had similarly high closeness centrality. The statistical significance of the differences between the centrality index values is shown in Supplementary Figure S10. Intention to drop out had closeness and strength centrality values that did not differ statistically from those of B1-2 and I1-2, although this node was more central than most of the other nodes in the network. The betweenness centrality of intention to drop out, however, was affected by sampling variability: This index did not differ from the rest of the nodes as clearly as the other centrality indices. This pattern of results can be interpreted such that intention to drop out was easily affected by the other nodes in the network and affected them in return, and that its connections to the other nodes of the network were quite strong. Nevertheless, whether the shortest paths between other pairs of nodes go through intention to drop out remains an open question.

## Discussion

The present study examined student engagement in education as a network of interlinked components, building on the theoretical conceptualization of engagement presented in Korhonen (2012) and Korhonen et al. (2017). The structure of the network model of engagement was remarkably similar to that of the theoretical conceptualization. Research question one about core components of engagement was confirmed in the results both theoretically and through the network model. According to the theoretical assumptions of the process model of engagement, successful long-term engagement builds on an emerging sense of belonging and an evolving identity as a university student (Korhonen, 2012; Korhonen et al., 2017). Theoretically, these overarching components of engagement bind together the remaining components of engagement: meaningfulness, participating in social practices and study skills. This is what we found in the network model: The nodes related to sense of belonging (B1, B2) and identity (I1, I2) figured as the most central ones in the network, connecting the remaining components. Interestingly, the centrality of the nodes in the network bore no obvious relationship to how commonly the corresponding statements were endorsed. For instance, the most commonly endorsed item M1 (“my education supports my self-development”) was among the least central nodes in the network, and the extremely infrequently endorsed item B2 (“I feel alienated from the university”) was among the most central nodes.

The present contribution can be considered substantive-methodological synergy (Borsboom, 2006; Marsh and Hau, 2007), in which a novel analysis method was used to address a substantively important research question based on a recently developed theory. In particular, student engagement is a dynamic phenomenon that emerges from the interaction of its components (Korhonen and Rautopuro, 2012, 2018), and network analysis is a method that is naturally suited to analyzing the dynamics in such systems (Boccaletti et al., 2006). Further, network models can assess the centrality of the components of a phenomenon (for the definition of the term “component,” see the section “Introduction”), unlike factor analysis that treats all observed variables as equally good indicators of the latent traits. Assessing the centrality of the items allows us to draw conclusions concerning the nature of the phenomenon of engagement that would not be possible based on factor analysis of the same data. For instance, when looking at the nodes related to the meaning of studies, node M1 (self-development) represents the cognitive aspect of meaning and is not particularly central in the network, whereas node M2 (enthusiasm) represents the emotional aspect of meaning and is a much more central node in the network. In the social practices scale item Pr1 (working in small groups) seemed to be positioned less centrally than compared to its counterpart item Pr2 (solitary enterprise), which raises questions about the sparsity of collaborative engaging practices in general.

In response to the research question two, a model including certain covariates of engagement, identified in previous studies (Korhonen and Rautopuro, 2012; Kahu, 2013; Millard et al., 2013), was presented. In the network model with covariates, personal-intellectual motivation shared a positive edge with enthusiasm (M2), which, in turn, shared a negative edge with intention to drop out. Thus, it can be hypothesized, based on the network model, that the relationship between personal-intellectual motivation and intention to drop out observed here and in Korhonen and Rautopuro’s (2012) study is mediated through enthusiasm (but see the “Limitations” section for a warning about over-interpreting the present results). Further, when student approaches to learning are investigated in the network model, the deep approach has a strong connection to personal-intellectual development (Pr-), which appears to mediate the relationship of the deep approach to nodes related to meaning, sense of belonging and identity. In addition, the direct connections between the deep approach and the cognitive component of meaningfulness (M1) and having found an appropriate study method (I2) are reminiscent of the way the deep approach is occasionally seen as part of cognitive engagement (Fredricks et al., 2004). The strategic approach is strongly connected to components of academic skills (Sk1, Sk2) and the other component of identity (having found an appropriate study method, I2).

The intention to drop out became one of the most central nodes in the network, with the strength and closeness centrality values exceeding those of the other nodes of the network. As the edges in psychometric network models can be interpreted as causal hypotheses (Borsboom, 2017; Epskamp et al., 2018), it is of interest to examine the connections between intention to drop out and the rest of the network in more detail. The negative edges from intention to drop out (Drp) to feelings of enthusiasm (meaning, M2) and certainty concerning the chosen field of study (Crt) suggest that enthusiasm and certainty protect students against intention to drop out, whereas feelings of alienation (belongingness, B2), the surface approach to learning (Srf) and default motivation (Dfl) function as predisposing factors. The result is in line with that of Korhonen and Rautopuro (2012) who found the personal-intellectual motivation and default motivation are similarly related to intention to drop out.

### Limitations of the Study

As noted above, it is a central assumption in psychometric network models that the nodes of the network correspond to components of the phenomenon under investigation. This may not be the case for all the nodes in network models. For instance, the strong edges connecting the strategic approach to learning with the items related to academic skills (Sk1 and Sk2) may be related to the fact that these phenomena were operationalized using similarly formulated questions. It may be that adding the node “strategic approach” to the network provides little unique information, over and above that included in the two skills-related nodes. Further, would the models have been robust to the replacement of the two practices-related items with ones that tap into other components of practices than the individual and collective aspects of engagement? Similar considerations apply to the choice of covariates, which may affect the relationships among the rest of the nodes. However, the relationships between the engagement-related nodes were quite robust to the addition of the covariates (the nodes did not essentially change with the addition of the covariates), which shows that the results reported in the first network model were not artifacts caused by the covariates added to the second model.

One obvious shortcoming of the present study is that we were not able to test the causal assumptions and hypotheses that were formed based on the two network models. This is because the present study was based on a cross-sectional sample instead of time-series data. Intensive longitudinal data, perhaps collected using the experience sampling method (ESM), might prove useful in making more educated guesses concerning the presence and direction of causal links among the components of engagement. This would enable us to think about causal effects at least in terms of Granger causality, which is based on the fulfilling the temporal requirement of causality, that is, the cause preceding the effect (Epskamp et al., 2018).

Finally, an important potential limitation of the network models must be discussed. It remains possible that some of the non-zero edges in the network models were artifacts due to conditioning on a collider variable. A collider is defined for a pair of variables as a third variable that is causally influenced by both. When one statistically or through experimental design controls for a collider variable, a spurious association may appear between the two variables. These ideas are introduced clearly and using intuitive examples in Rohrer (2018) in an article that considers problems inherent in drawing causal inferences based on correlational data. Although it is possible that in the future novel statistical methods might allow researchers to diagnose such situations, for now the advice given to applied researchers is to use their own judgment in choosing the relevant variables to include in a network model and to not over-interpret the results as unproblematically representing reality (Fried and Cramer, 2017). In the present covariate model, the negative edge between personal-intellectual motivation and certainty concerning one’s field of study may be a candidate for a spurious edge that is due to conditioning on a collider. It is possible that the two would function as causes of M1 (education supports self-development); That is, the more one emphasizes personal growth as a motivation for studying and the more certain one is about one’s chosen field of study, the more one comes to think that one’s education supports one’s self-development. When we inspect the pattern of zero-order correlations (Supplementary Table S3) against the network model, we notice that the correlation between personal-intellectual motivation and certainty is practically zero. The triangle formed between Pa1, Pa2, and Pr2 is another candidate for potential spurious associations, but when we examine the zero-order correlations (Supplementary Table S3) among these variables against the network model (or its adjacency matrix in Supplementary Table S4), we notice that they are both of an equal sign, with the zero-order correlations just slightly stronger. To conclude, it cannot be ruled out that the former of these examples could be due to conditioning on a collider, whereas we do not believe the latter is an example of a spurious result.

### Implications for Higher Education and Suggestions for Further Studies

The unique benefit of this analytical approach was that by calculating different centrality indices we quantified the importance of the individual components of engagement. This is in marked contrast to previous studies of student engagement (for a comprehensive review, see Kahu, 2013), which conceptualized the dimensions of engagement as latent variables and thus, perceived the observed variables as equally good indicators of the latent dimensions, differing only in the amount of error variance of each observed variable.

Thus, based on the present findings, we can make certain novel practical recommendations concerning interventions for students who struggle with engagement during their first year of higher education. First, if a student feels alienated from the university, this should be an immediate warning sign of potential problems in other areas of engagement, such as skills, practices, and participation. In practice, feelings of alienation could be targeted by, for example, providing low-threshold access to education psychologists and encouraging students to contact the professionals immediately if the students experience feelings of alienation. Second, it is important to encourage first-year students to consider from the very beginning of their education an appropriate study method. For instance, planning systematically interactive first-year courses and intensive peer-group activities and including discussion groups that focus on different study methods might encourage first-year students to think about the aspects of engagement that are the best for them. In addition, by early identification of drop-out intentions or shortcomings in academic skills, it can be influenced the students to correct wrong study field choices or identify not being capable of studying in higher education. This can have a very positive meaning for the student personally.

In addition, using the model with covariates, we formulated causal hypotheses and tentative recommendations concerning preventing students from dropping out of education. It has been observed that most dropouts leave their study program or university during their first year (Rautopuro and Väisänen, 2001; Korhonen and Rautopuro, 2018). This phenomenon seems to be stable in Finnish higher education, despite the many reforms implemented over the years (Korhonen and Rautopuro, 2018). As noted above, in the present study alienation (B2) seems to be a central mediating node between intention to drop out (Drp) and the nodes related to participation (Pa1, Pa2), as well as between dropping out and “belongingness” (B1). This may indicate that if one wants to prevent experiences of alienation and support engagement and feelings of belonging, it is necessary to create such educational environments for teaching and learning where it is natural to participate and work together. Further, we propose that an increase in alienation, a particularly central experience in the engagement network, will have a larger effect on the other components of engagement than an increase in a more peripheral experience, such as knowing other students (Pr1). In particular, studies focusing on individual-level networks of engagement in a time-series design (i.e., separate network models for individual students) would enable us to examine phase transitions from the engaged to the disengaged state. This process might share essential similarities with the proposed phase transition from the non-depressed to depressed state as examined in network models of psychopathology (Borsboom, 2017; Fried et al., 2017).

In addition, we hypothesize, based on the covariate model, that an intervention that increases students’ enthusiasm (M2) for education will decrease their intention to drop out (other things being equal). However, an increase in the perception that education supports one’s self-development (M1), may exert influence intention to drop out unless they also become more enthusiastic about their education (M2) and more certain that their field of study is the correct choice for them (i.e., the effect of the cognitive component of meaningfulness may exert influence intention to drop out through other phenomena). In short, network analysis provides interesting hypotheses to test in future studies.

A further research topic for the application of network analysis and models of engagement could be to consider the time dimension and in what ways the engagement phenomenon develops over a certain time period. For instance, when on a macro-level (institutionally or cross-institutionally) is monitored the same student cohort and their engagement with periodical measurements (Korhonen et al., 2017). Alternatively, in the micro-level case (selected sample of students), when the ESM is used as a structured diary method (Verhagen et al., 2016) and subjective experiences are assessed daily during the follow-up period. It would be interesting to more closely follow whether central nodes in the network work well for predictors for strong engagement or intention to drop out. All these options assume diverse follow-up study designs.

## Data Availability

The datasets generated for this study are available on request to the corresponding author.

## Ethics Statement

This study was carried out according to the ethical guidelines of the Finnish National Board of Research Integrity (https://www.tenk.fi/en) for this kind of research in the Finnish research context. All participants received written informed consent in accordance with the guidelines.

## Author Contributions

VK, AT, MI, and MM: conceptualization. VK, AT, and MI: investigation and funding. VK and AT: project management. AT: resources. MM: methodology, software, analysis, validation, and visualization. VK, MM, and AT: original draft preparation and final editing.

## Conflict of Interest Statement

The authors declare that the research was conducted in the absence of any commercial or financial relationships that could be construed as a potential conflict of interest.
