# Supplementary material for: Understanding the Multidimensional Nature of Student Engagement During the First Year of Higher Education
Source: Front Psychol. 2019 May 10;10:1056. doi: 10.3389/fpsyg.2019.01056 (PMC6524002; doi:10.3389/fpsyg.2019.01056)
Supplement: Supplementary file 1 [file Data_Sheet_1.pdf]

Understanding the multidimensional nature of student engagement during the first year of higher education

Vesa Korhonen[1], Markus Mattsson[2], Mikko Inkinen[2] & Auli Toom[2]

[1]Faculty of Education and Culture, Tampere University, Tampere, Finland
[2] Centre for University Teaching and Learning, Faculty of Educational Sciences, University of Helsinki, Finland

Supplementary materials

Supplementary table S1 Correlation matrix for the network model of engagement

Variable	M1	M2	Pa1	Pa2	Pr1	Pr2	Sk1	Sk2	I1	I2	B1	B2
M1	1.00	0.72	-0.15	0.17	0.13	-0.03	0.21	0.32	0.33	0.36	0.39	-0.31
M2	0.72	1.00	-0.24	0.26	0.14	-0.11	0.27	0.38	0.47	0.46	0.55	-0.46
Pa1	-0.15	-0.24	1.00	-0.63	-0.10	0.31	0.02	-0.06	-0.14	-0.10	-0.25	0.48
Pa2	0.17	0.26	-0.63	1.00	0.14	-0.30	0.02	0.10	0.19	0.14	0.31	-0.46
Pr1	0.13	0.14	-0.10	0.14	1.00	-0.32	0.04	0.07	0.08	0.14	0.10	-0.10
Pr2	-0.03	-0.11	0.31	-0.30	-0.32	1.00	0.12	0.09	-0.06	-0.04	-0.14	0.28
Sk1	0.21	0.27	0.02	0.02	0.04	0.12	1.00	0.54	0.28	0.47	0.17	-0.10
Sk2	0.32	0.38	-0.06	0.10	0.07	0.09	0.54	1.00	0.39	0.50	0.25	-0.20
I1	0.33	0.47	-0.14	0.19	0.08	-0.06	0.28	0.39	1.00	0.60	0.64	-0.38
I2	0.36	0.46	-0.10	0.14	0.14	-0.04	0.47	0.50	0.60	1.00	0.44	-0.30
B1	0.39	0.55	-0.25	0.31	0.10	-0.14	0.17	0.25	0.64	0.44	1.00	-0.55
B2	-0.31	-0.46	0.48	-0.46	-0.10	0.28	-0.10	-0.20	-0.38	-0.30	-0.55	1.00

Supplementary table S2 Adjacency matrix for the network model of engagement

Variable	M1	M2	Pa1	Pa2	Pr1	Pr2	Sk1	Sk2	I1	I2	B1	B2
M1	0.00	0.58	0.00	0.00	0.04	0.00	0.00	0.05	0.00	0.03	0.00	0.00
M2	0.58	0.00	0.00	0.02	0.00	0.00	0.01	0.08	0.04	0.11	0.20	-0.15
Pa1	0.00	0.00	0.00	-0.48	0.00	0.11	0.02	0.00	0.00	0.00	0.00	0.23
Pa2	0.00	0.02	-0.48	0.00	0.01	-0.10	0.00	0.00	0.00	0.00	0.05	-0.14
Pr1	0.04	0.00	0.00	0.01	0.00	-0.27	0.00	0.00	0.00	0.06	0.00	0.00
Pr2	0.00	0.00	0.11	-0.10	-0.27	0.00	0.07	0.06	0.00	0.00	0.00	0.11
Sk1	0.00	0.01	0.02	0.00	0.00	0.07	0.00	0.36	0.00	0.23	0.00	0.00
Sk2	0.05	0.08	0.00	0.00	0.00	0.06	0.36	0.00	0.07	0.19	0.00	0.00
I1	0.00	0.04	0.00	0.00	0.00	0.00	0.00	0.07	0.00	0.35	0.42	0.00
I2	0.03	0.11	0.00	0.00	0.06	0.00	0.23	0.19	0.35	0.00	0.00	-0.02
B1	0.00	0.20	0.00	0.05	0.00	0.00	0.00	0.00	0.42	0.00	0.00	-0.28
B2	0.00	-0.15	0.23	-0.14	0.00	0.11	0.00	0.00	0.00	-0.02	-0.28	0.00

Supplementary table S3 Correlation matrix for the network model of engagement and covariates

Variable	M1	M2	Pa1	Pa2	Pr1	Pr2	Sk1	Sk2	I1	I2	B1	B2	Pers-int	Def	Cert	Drp	Deep	Strat	S
M1	1.00	0.72	-0.15	0.17	0.14	-0.03	0.21	0.32	0.33	0.37	0.39	-0.31	0.40	-0.40	0.35	-0.44	0.32	0.25	-0
M2	0.72	1.00	-0.24	0.26	0.14	-0.11	0.27	0.38	0.47	0.46	0.55	-0.47	0.44	-0.51	0.33	-0.58	0.31	0.33	-0
Pa1	-0.15	-0.24	1.00	-0.63	-0.10	0.31	0.02	-0.06	-0.14	-0.10	-0.25	0.49	-0.04	0.19	-0.08	0.27	0.04	-0.04	0
Pa2	0.17	0.26	-0.63	1.00	0.15	-0.30	0.02	0.10	0.19	0.14	0.31	-0.46	0.09	-0.14	0.05	-0.24	0.05	0.08	0
Pr1	0.14	0.14	-0.10	0.15	1.00	-0.32	0.05	0.07	0.07	0.14	0.10	-0.10	0.05	-0.07	0.07	-0.10	0.10	0.09	-0
Pr2	-0.03	-0.11	0.31	-0.30	-0.32	1.00	0.12	0.09	-0.06	-0.04	-0.14	0.28	0.03	0.11	0.02	0.19	0.08	0.02	0
Sk1	0.21	0.27	0.02	0.02	0.05	0.12	1.00	0.54	0.28	0.47	0.17	-0.10	0.20	-0.18	0.18	-0.19	0.27	0.63	-0
Sk2	0.32	0.38	-0.06	0.10	0.07	0.09	0.54	1.00	0.39	0.50	0.25	-0.20	0.25	-0.24	0.18	-0.27	0.31	0.57	-0
I1	0.33	0.47	-0.14	0.19	0.07	-0.06	0.28	0.39	1.00	0.60	0.65	-0.38	0.44	-0.38	0.12	-0.41	0.28	0.40	-0
I2	0.37	0.46	-0.10	0.14	0.14	-0.04	0.47	0.50	0.60	1.00	0.44	-0.31	0.30	-0.32	0.21	-0.39	0.35	0.58	-0
B1	0.39	0.55	-0.25	0.31	0.10	-0.14	0.17	0.25	0.65	0.44	1.00	-0.55	0.44	-0.43	0.14	-0.46	0.21	0.23	-0
B2	-0.31	-0.47	0.49	-0.46	-0.10	0.28	-0.10	-0.20	-0.38	-0.31	-0.55	1.00	-0.20	0.40	-0.15	0.48	-0.08	-0.20	0
Pers-int	0.40	0.44	-0.04	0.09	0.05	0.03	0.20	0.25	0.44	0.30	0.44	-0.20	1.00	-0.29	0.02	-0.24	0.41	0.23	-0
Def	-0.40	-0.51	0.19	-0.14	-0.07	0.11	-0.18	-0.24	-0.38	-0.32	-0.43	0.40	-0.29	1.00	-0.17	0.51	-0.17	-0.23	0
Cert	0.35	0.33	-0.08	0.05	0.07	0.02	0.18	0.18	0.12	0.21	0.14	-0.15	0.02	-0.17	1.00	-0.32	0.06	0.17	-0
Drp	-0.44	-0.58	0.27	-0.24	-0.10	0.19	-0.19	-0.27	-0.41	-0.39	-0.46	0.48	-0.24	0.51	-0.32	1.00	-0.12	-0.25	0
Deep	0.32	0.31	0.04	0.05	0.10	0.08	0.27	0.31	0.28	0.35	0.21	-0.08	0.41	-0.17	0.06	-0.12	1.00	0.35	-0
Strat	0.25	0.33	-0.04	0.08	0.09	0.02	0.63	0.57	0.40	0.58	0.23	-0.20	0.23	-0.23	0.17	-0.25	0.35	1.00	-0
Surf	-0.19	-0.23	0.04	0.00	-0.05	0.09	-0.11	-0.11	-0.29	-0.37	-0.17	0.20	-0.10	0.26	-0.15	0.31	-0.21	-0.22	1

Supplementary table S4 Adjacency matrix for the network model of engagement and covariates

Variable	M1	M2	Pa1	Pa2	Pr1	Pr2	Sk1	Sk2	I1	I2	B1	B2	Pers-int	Def	Cert	Drp	Deep	Strat	S
M1	0.00	0.49	0.00	0.00	0.03	0.00	0.00	0.03	0.00	0.00	0.00	0.00	0.10	-0.03	0.17	-0.01	0.10	0.00	0
M2	0.49	0.00	0.00	0.02	0.00	0.00	0.00	0.08	0.00	0.07	0.14	-0.07	0.10	-0.14	0.05	-0.21	0.00	0.00	0
Pa1	0.00	0.00	0.00	-0.48	0.00	0.11	0.01	0.00	0.00	0.00	0.00	0.22	0.02	0.00	0.00	0.02	0.04	0.00	0
Pa2	0.00	0.02	-0.48	0.00	0.02	-0.10	0.00	0.00	0.00	0.00	0.05	-0.14	0.00	0.00	0.00	0.00	0.00	0.00	0
Pr1	0.03	0.00	0.00	0.02	0.00	-0.27	0.00	0.00	0.00	0.05	0.00	0.00	0.00	0.00	0.00	0.00	0.03	0.00	0
Pr2	0.00	0.00	0.11	-0.10	-0.27	0.00	0.06	0.05	0.00	0.00	0.00	0.09	0.03	0.00	0.02	0.05	0.04	0.00	0
Sk1	0.00	0.00	0.01	0.00	0.00	0.06	0.00	0.23	0.00	0.09	-0.01	0.00	0.00	0.00	0.05	0.00	0.01	0.39	0
Sk2	0.03	0.08	0.00	0.00	0.00	0.05	0.23	0.00	0.05	0.13	0.00	0.00	0.00	0.00	0.01	-0.01	0.05	0.22	0
I1	0.00	0.00	0.00	0.00	0.00	0.00	0.00	0.05	0.00	0.30	0.38	0.00	0.14	-0.05	0.00	-0.03	0.00	0.03	-0
I2	0.00	0.07	0.00	0.00	0.05	0.00	0.09	0.13	0.30	0.00	0.00	0.00	0.00	0.00	0.00	-0.05	0.07	0.24	-0
B1	0.00	0.14	0.00	0.05	0.00	0.00	-0.01	0.00	0.38	0.00	0.00	-0.26	0.13	-0.06	0.00	-0.03	0.00	0.00	0
B2	0.00	-0.07	0.22	-0.14	0.00	0.09	0.00	0.00	0.00	0.00	-0.26	0.00	0.00	0.08	0.00	0.16	0.01	0.00	0
Pers-int	0.10	0.10	0.02	0.00	0.00	0.03	0.00	0.00	0.14	0.00	0.13	0.00	0.00	-0.02	-0.10	0.00	0.25	0.00	0
Def	-0.03	-0.14	0.00	0.00	0.00	0.00	0.00	0.00	-0.05	0.00	-0.06	0.08	-0.02	0.00	0.00	0.22	0.00	0.00	0
Cert	0.17	0.05	0.00	0.00	0.00	0.02	0.05	0.01	0.00	0.00	0.00	0.00	-0.10	0.00	0.00	-0.14	0.00	0.00	-0
Drp	-0.01	-0.21	0.02	0.00	0.00	0.05	0.00	-0.01	-0.03	-0.05	-0.03	0.16	0.00	0.22	-0.14	0.00	0.03	0.00	0
Deep	0.10	0.00	0.04	0.00	0.03	0.04	0.01	0.05	0.00	0.07	0.00	0.01	0.25	0.00	0.00	0.03	0.00	0.11	-0
Strat	0.00	0.00	0.00	0.00	0.00	0.00	0.39	0.22	0.03	0.24	0.00	0.00	0.00	0.00	0.00	0.00	0.11	0.00	0
Surf	0.00	0.00	0.00	0.04	0.00	0.03	0.00	0.04	-0.03	-0.19	0.00	0.00	0.02	0.07	-0.01	0.12	-0.07	0.00	0

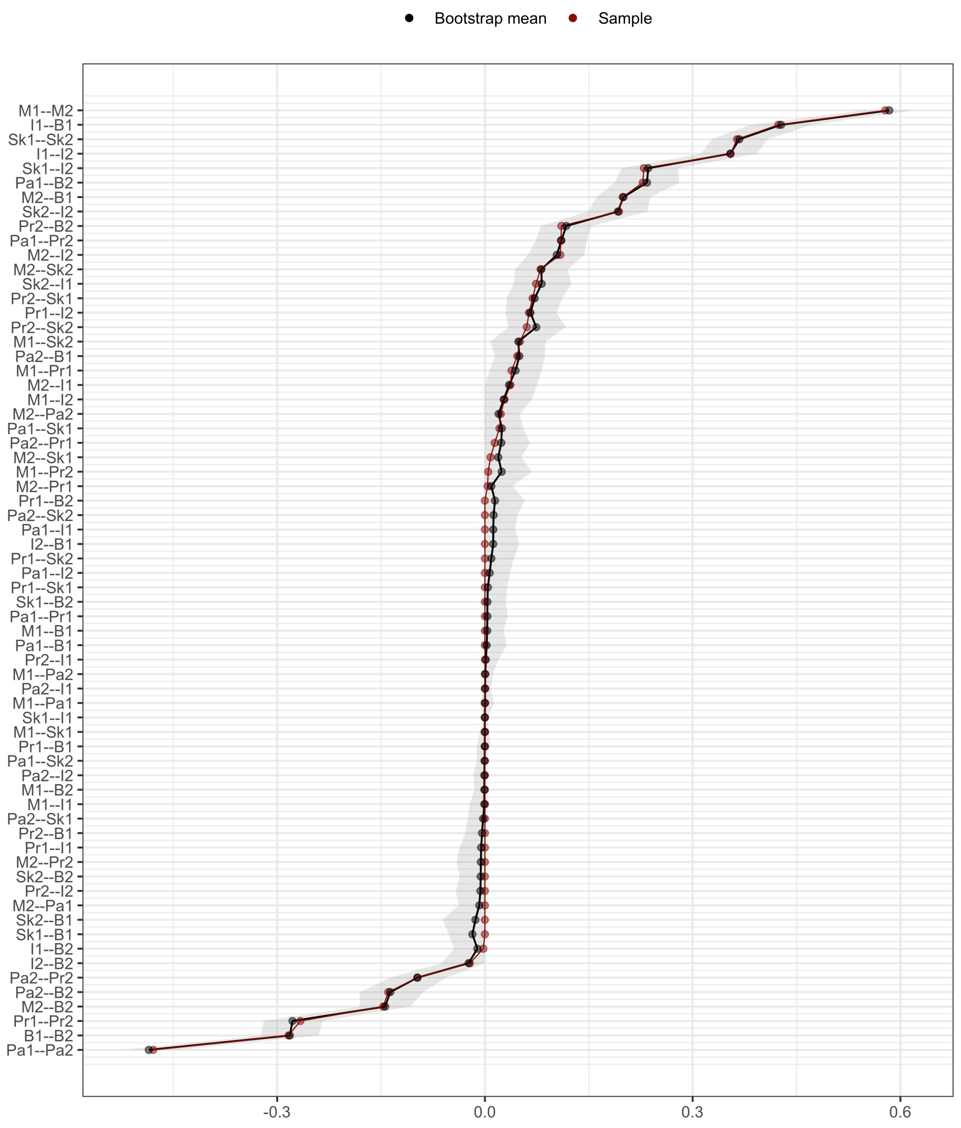

Figure S1. The bootstrapped confidence intervals of the edge weights of the network model of student engagement.

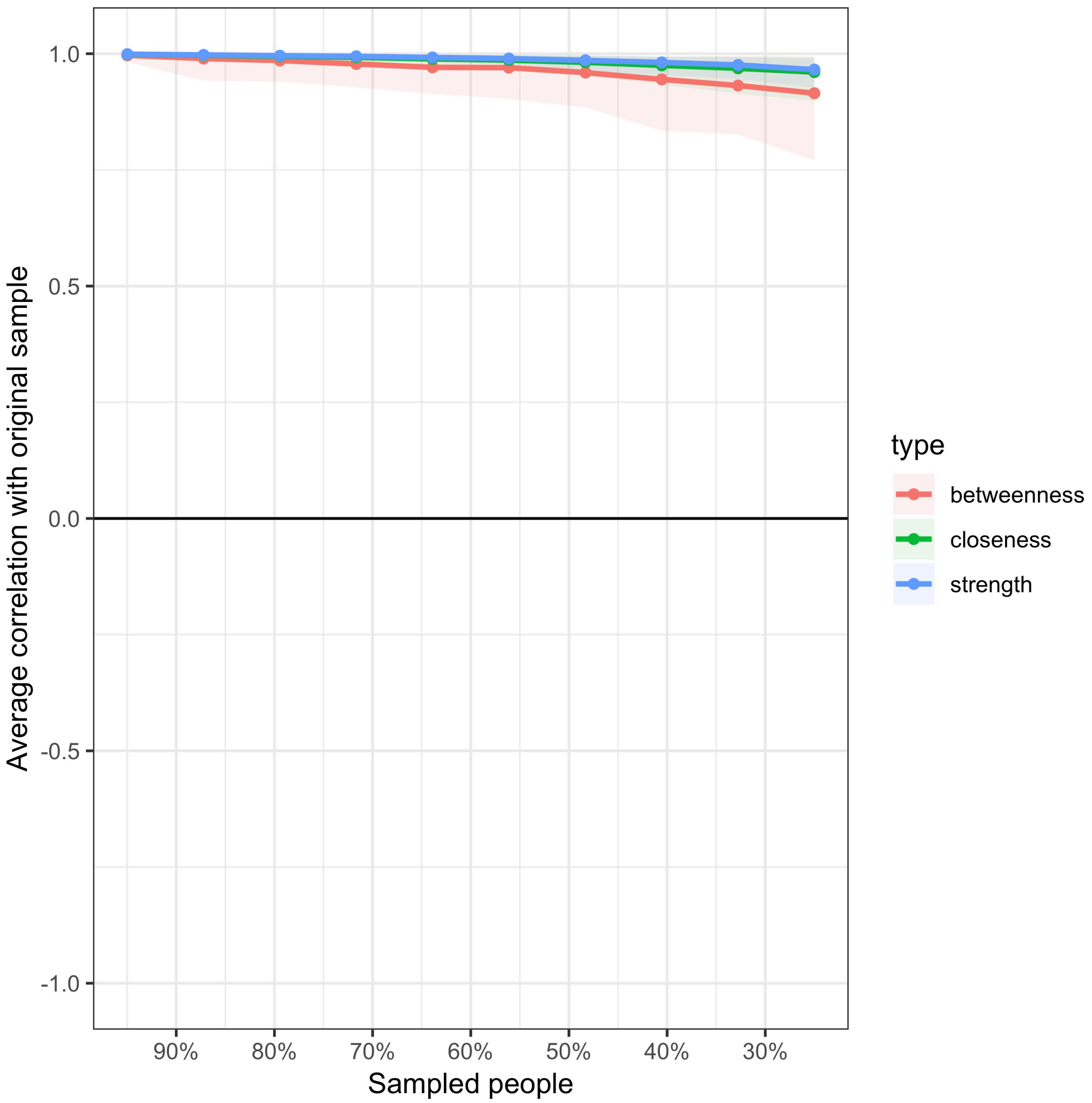

Figure S2. The stability of the centrality indices of the network model of student engagement.

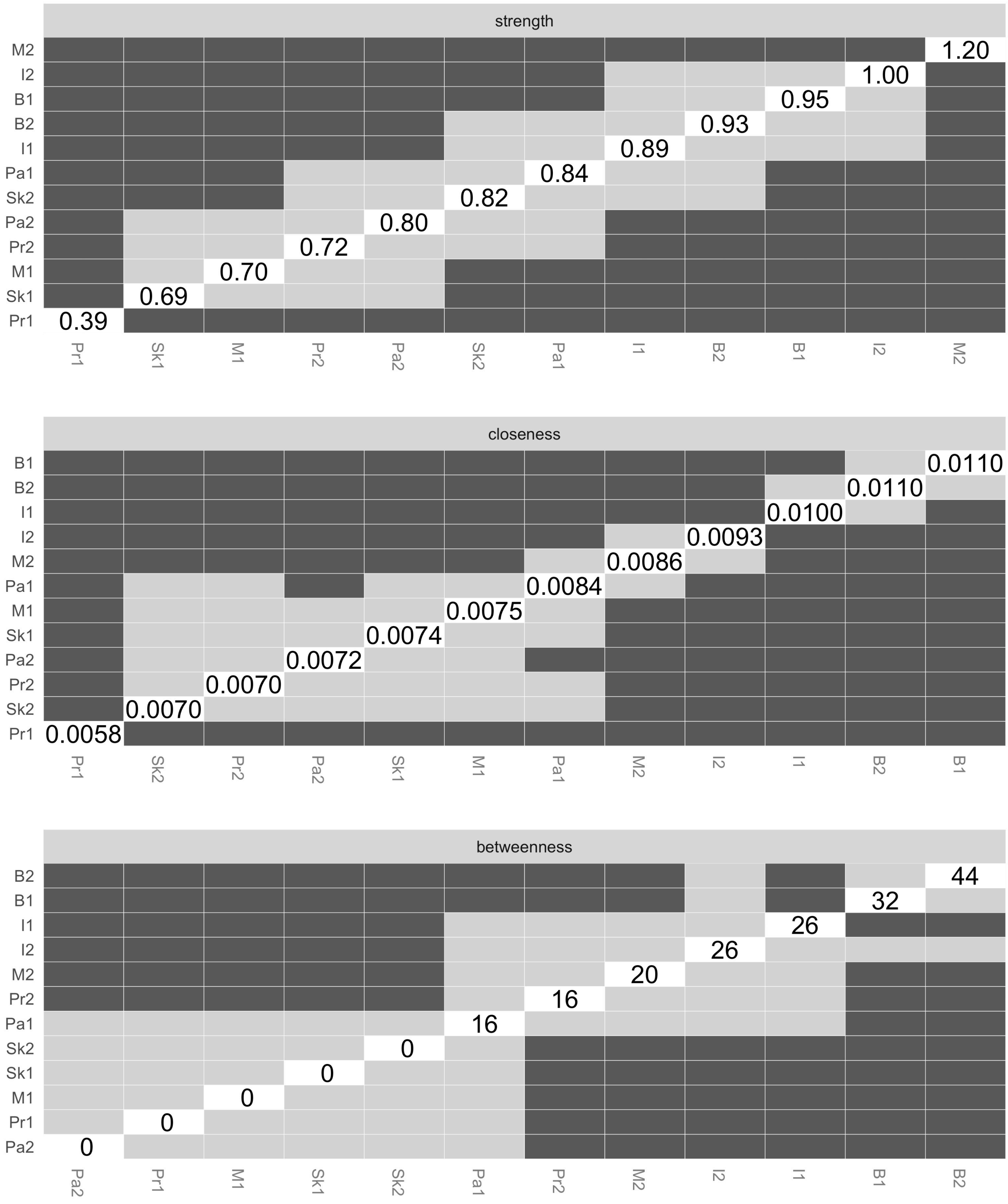

Figure S3. Bootstrapped difference tests (α = 0.05) of the centrality values of the 12 engagement nodes in the engagement network. The values on the diagonals indicate the non-standardized values of the centrality coefficients, light gray squares indicate non-significant differences among the centrality values of the nodes, and dark gray squares indicate significant differences.

Analysis with covariates

Variable	M1	M2	Pa1	Pa2	Pr1	Pr2	Sk1	Sk2	I1	I2	B1	B2	Pers-int	Def	Cert	Drp	Deep	Strat	Surf
M1	0.00	0.49	0.00	0.00	0.03	0.00	0.00	0.03	0.00	0.00	0.00	0.00	0.10	-0.03	0.17	-0.01	0.10	0.00	0.00
M2	0.49	0.00	0.00	0.02	0.00	0.00	0.00	0.08	0.00	0.07	0.14	-0.07	0.10	-0.14	0.05	-0.21	0.00	0.00	0.00
Pa1	0.00	0.00	0.00	-0.48	0.00	0.11	0.01	0.00	0.00	0.00	0.00	0.22	0.02	0.00	0.00	0.02	0.04	0.00	0.00
Pa2	0.00	0.02	-0.48	0.00	0.02	-0.10	0.00	0.00	0.00	0.00	0.05	-0.14	0.00	0.00	0.00	0.00	0.00	0.00	0.04
Pr1	0.03	0.00	0.00	0.02	0.00	-0.27	0.00	0.00	0.00	0.05	0.00	0.00	0.00	0.00	0.00	0.00	0.03	0.00	0.00
Pr2	0.00	0.00	0.11	-0.10	-0.27	0.00	0.06	0.05	0.00	0.00	0.00	0.09	0.03	0.00	0.02	0.05	0.04	0.00	0.03
Sk1	0.00	0.00	0.01	0.00	0.00	0.06	0.00	0.23	0.00	0.09	-0.01	0.00	0.00	0.00	0.05	0.00	0.01	0.39	0.00
Sk2	0.03	0.08	0.00	0.00	0.00	0.05	0.23	0.00	0.05	0.13	0.00	0.00	0.00	0.00	0.01	-0.01	0.05	0.22	0.04
I1	0.00	0.00	0.00	0.00	0.00	0.00	0.00	0.05	0.00	0.30	0.38	0.00	0.14	-0.05	0.00	-0.03	0.00	0.03	-0.03
I2	0.00	0.07	0.00	0.00	0.05	0.00	0.09	0.13	0.30	0.00	0.00	0.00	0.00	0.00	0.00	-0.05	0.07	0.24	-0.19
B1	0.00	0.14	0.00	0.05	0.00	0.00	-0.01	0.00	0.38	0.00	0.00	-0.26	0.13	-0.06	0.00	-0.03	0.00	0.00	0.00
B2	0.00	-0.07	0.22	-0.14	0.00	0.09	0.00	0.00	0.00	0.00	-0.26	0.00	0.00	0.08	0.00	0.16	0.01	0.00	0.00
Pers-int	0.10	0.10	0.02	0.00	0.00	0.03	0.00	0.00	0.14	0.00	0.13	0.00	0.00	-0.02	-0.10	0.00	0.25	0.00	0.02
Def	-0.03	-0.14	0.00	0.00	0.00	0.00	0.00	0.00	-0.05	0.00	-0.06	0.08	-0.02	0.00	0.00	0.22	0.00	0.00	0.07
Cert	0.17	0.05	0.00	0.00	0.00	0.02	0.05	0.01	0.00	0.00	0.00	0.00	-0.10	0.00	0.00	-0.14	0.00	0.00	-0.01
Drp	-0.01	-0.21	0.02	0.00	0.00	0.05	0.00	-0.01	-0.03	-0.05	-0.03	0.16	0.00	0.22	-0.14	0.00	0.03	0.00	0.12
Deep	0.10	0.00	0.04	0.00	0.03	0.04	0.01	0.05	0.00	0.07	0.00	0.01	0.25	0.00	0.00	0.03	0.00	0.11	-0.07
Strat	0.00	0.00	0.00	0.00	0.00	0.00	0.39	0.22	0.03	0.24	0.00	0.00	0.00	0.00	0.00	0.00	0.11	0.00	0.00
Surf	0.00	0.00	0.00	0.04	0.00	0.03	0.00	0.04	-0.03	-0.19	0.00	0.00	0.02	0.07	-0.01	0.12	-0.07	0.00	0.00

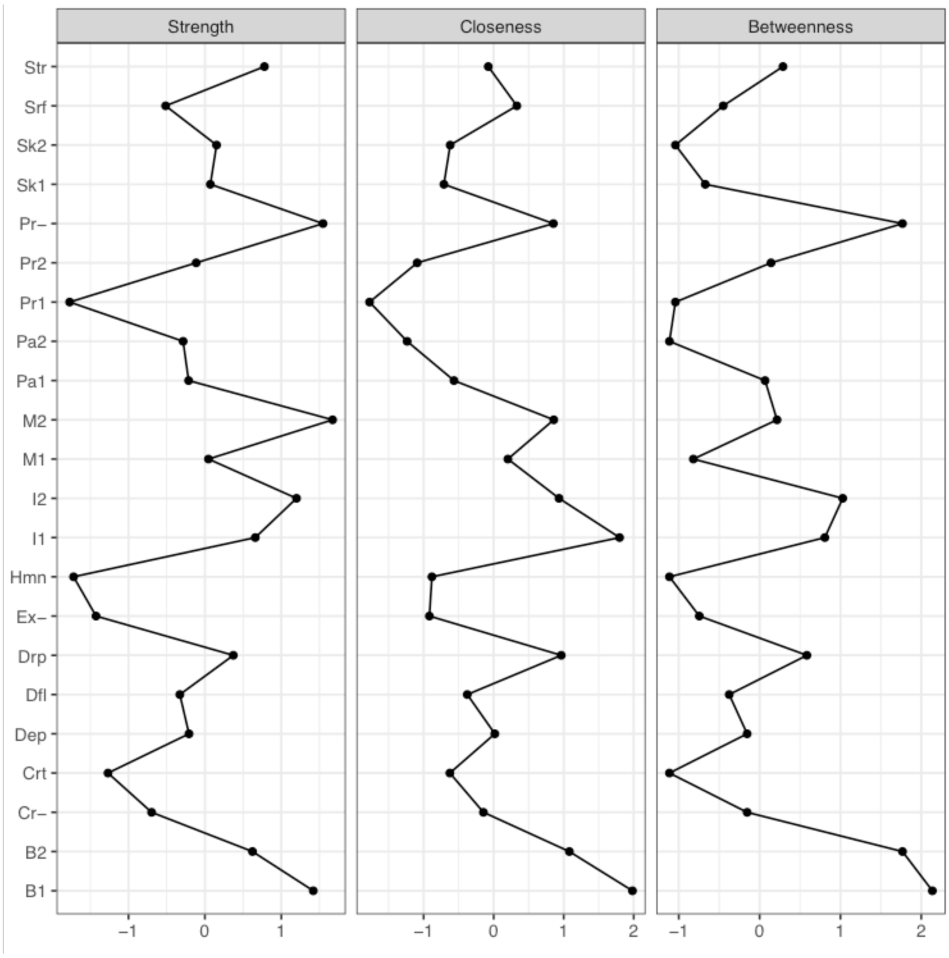

Figure S4. The network model of engagement and all the covariates.

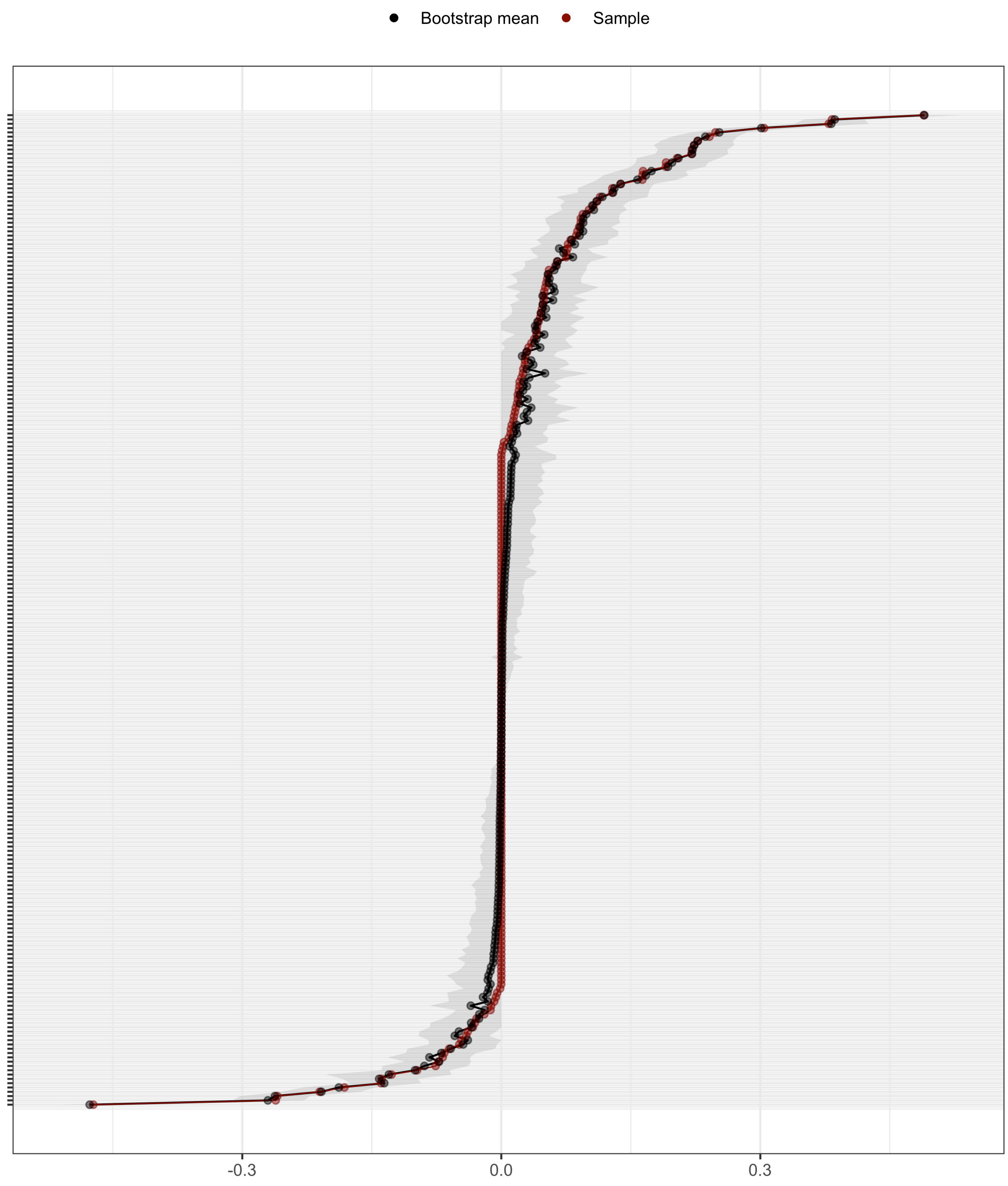

Figure S5. Bootstrapped 95 % confidence intervals for the network model of student engagement and the covariates (Figure S4).

The values of centrality indices were again quite stable (Figure S6), with all three centrality indices having CS-index values of 0.75.

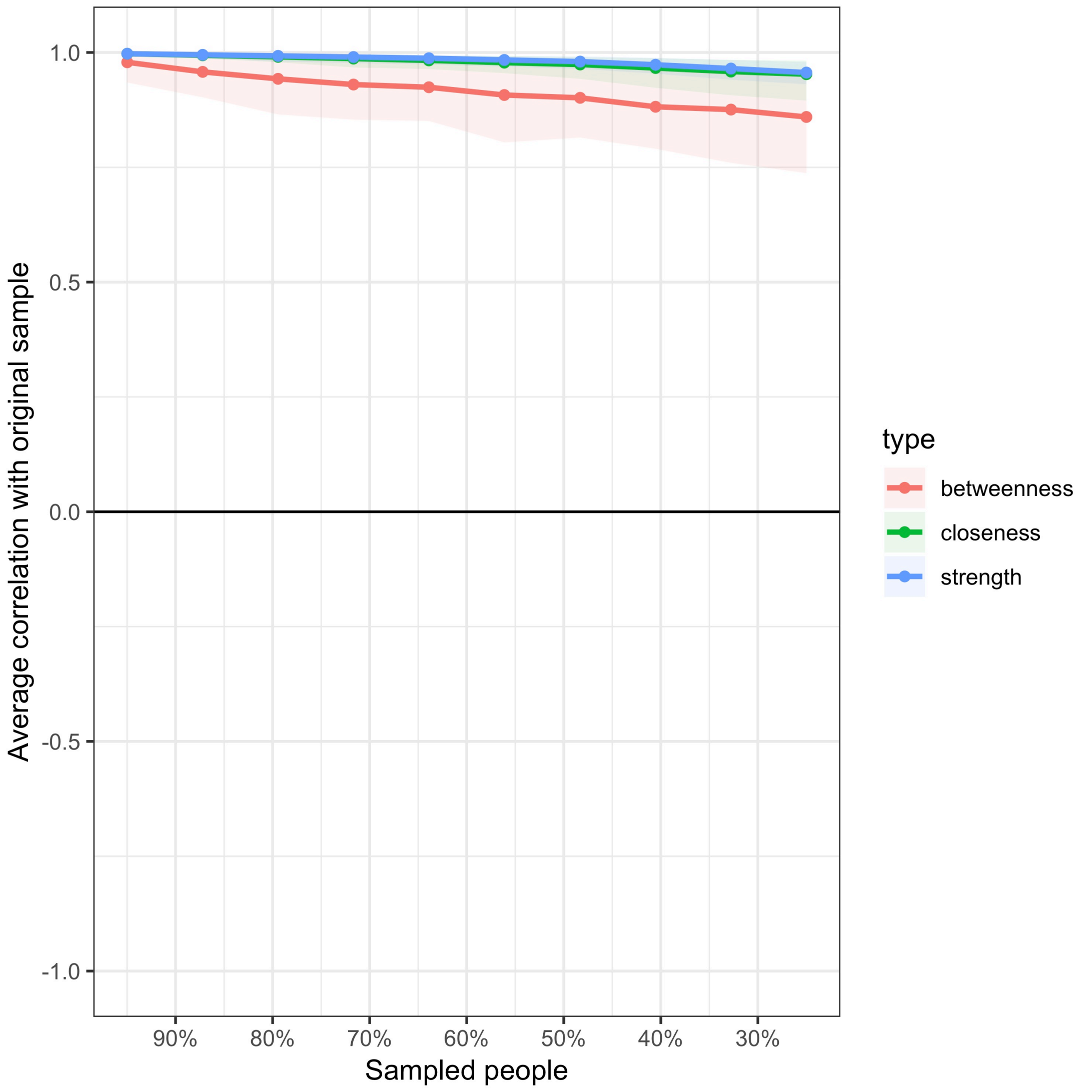

Figure S6. *The stability of the centrality indices of the network model of student engagement and the covariates (Figure S4).*

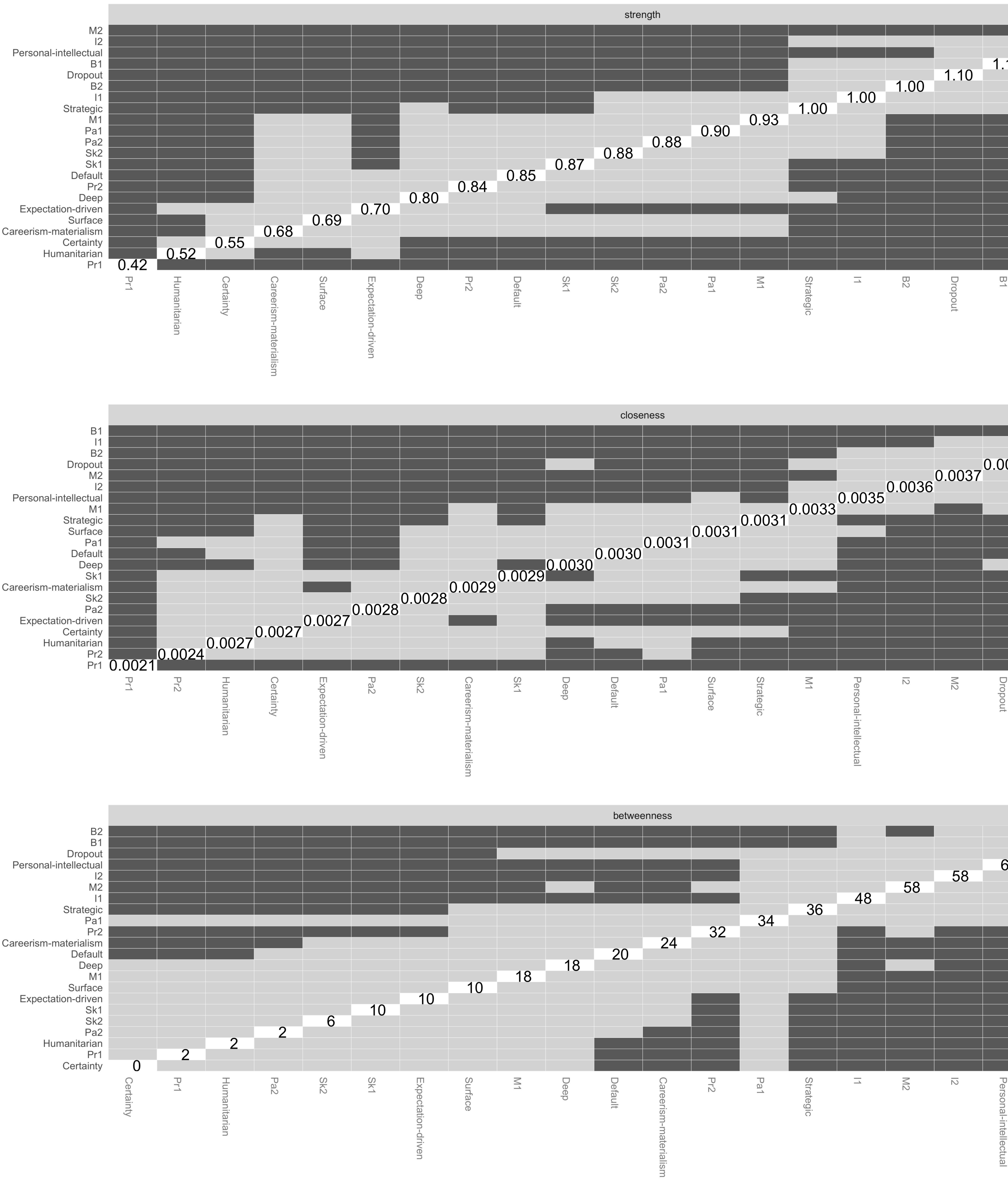

Figure S7. Differences among the values of the centrality indices for the network model of student engagement and the covariates (Figure S4).

Supplementary analyses involving the covariates reported in the main manuscript

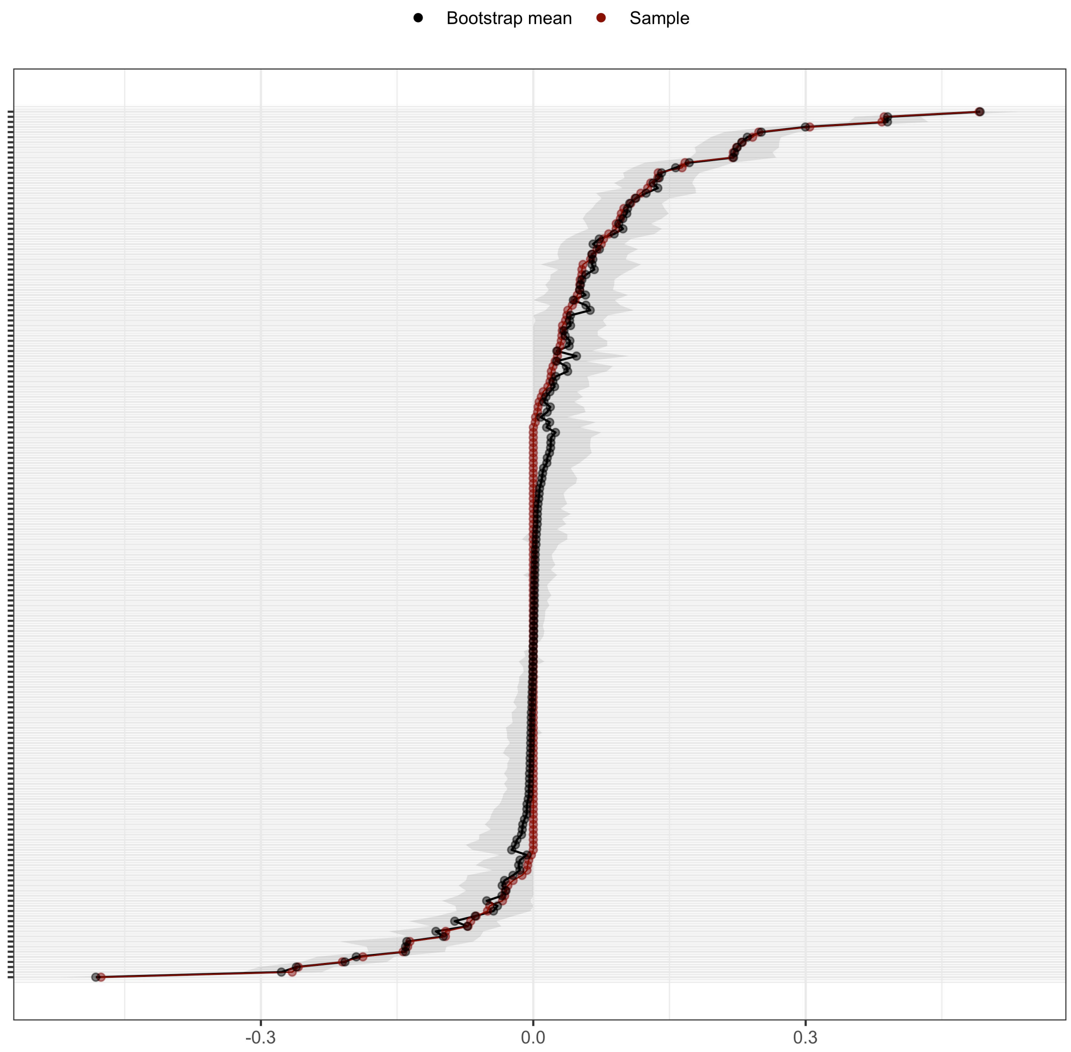

Figure S8. Bootstrapped 95 % confidence intervals for the network model of the engagement items and covariates (manuscript Figure 3).

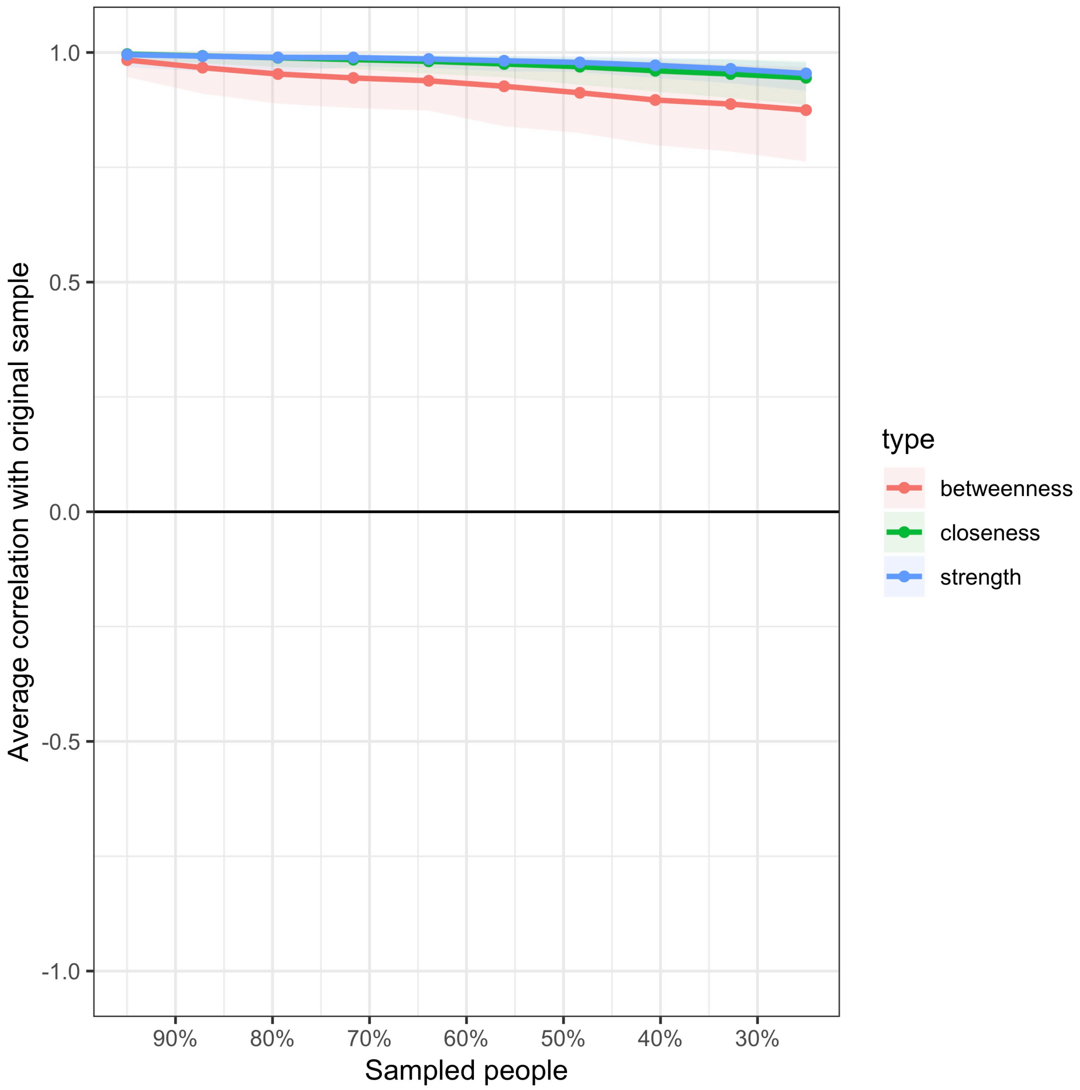

Figure S9. The stability of the centrality indices for the network model of the engagement items and covariates (manuscript Figure 3).

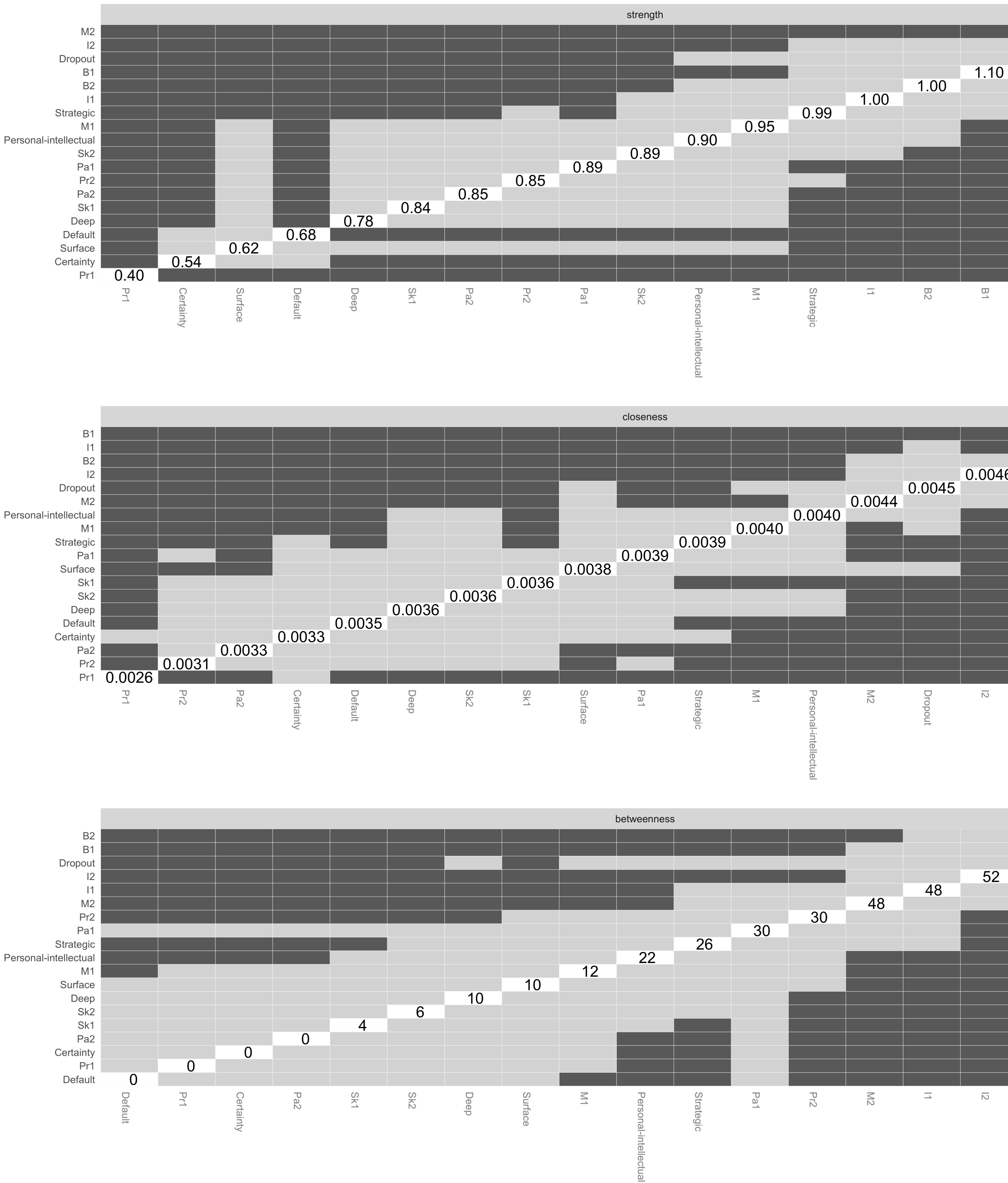

Figure S10. Differences among the values of the centrality indices for the network model of the engagement items and covariates (manuscript Figure 3).
